# The anti-ferroptotic role of FSP1: current molecular mechanism and therapeutic approach

**DOI:** 10.1186/s43556-022-00105-z

**Published:** 2022-11-29

**Authors:** Furong Zeng, Xiang Chen, Guangtong Deng

**Affiliations:** 1 Department of Oncology, Xiangya Hospital, Central South University, Changsha, China; 2 Department of Dermatology, Xiangya Hospital, Central South University, Changsha, China

A recent article by Mishima et al. in *Nature* demonstrated another mechanism of ferroptosis suppressor protein 1 (FSP1)-mediated ferroptosis resistance [1], motivating to summarize the anti-ferroptotic role of FSP1.

Ferroptosis is a form of regulated cell death characterized by iron-dependent accumulation of harmful lipid peroxides. Glutathione peroxidase 4 (GPX4), the central regulator that contracts ferroptosis, converts lipid hydroperoxides into non-toxic lipid alcohols in concert with glutathione [2]. However, GPX4 inhibition has failed to trigger ferroptosis in several cancer cell lines, indicating the existence of an alternative mechanism of ferroptosis resistance. Using unbiased genetic screens, FSP1 has been identified as a novel ferroptosis suppressor protein and a second mainstay of ferroptosis control after GPX4 [3, 4].

FSP1 was previously called flavoprotein apoptosis-inducing factor mitochondria-associated 2 (AIFM2). It was renamed FSP1 for its anti-ferroptotic role. FSP1 mainly localizes to the periphery of lipid droplets and to the plasma membrane. It also partly overlaps with the endoplasmic reticulum and Golgi. Bersuker et al. and Doll et al. independently reported that N-myristylation of FSP1 is essential for its anti-ferroptotic activity [3, 4]. Bersuker et al. demonstrated that N-myristylation-dependent recruitment of FSP1 to the plasma membrane promotes resistance to ferroptosis. Once recruited, FSP1 suppresses lipid hydroperoxides by catalyzing the reduction of ubiquinone (CoQ_10_) to its hydroquinone form, ubiquinol (CoQ_10_-H_2_), and the consumption of NAD(P)H [3]. These findings were further validated through co-autoxidation experiments with egg phosphatidylcholine and STY-BODIPY, using a lipophilic alkoxyl radical generator. In addition, considering the ready autoxidation of CoQ_10_-H_2_, α-tocopherol could either be reproduced indirectly via CoQ_10_-H_2_ or directly in vitro by FSP1 to function as a reactive radical-trapping antioxidant [4]. Interestingly, through inhibiting CoQ biosynthesis enzyme COQ2 with 4-chlorobenzoic acid (4-CBA) or knocking out COQ2, acute reduction of cellular CoQ levels exerts a lower degree of sensitization to RSL3 than FSP1 knockout [3]. This suggests that FSP1 mediated mechanisms other than the FSP1-CoQ_10_-NAD(P)H pathway may contribute to ferroptosis resistance.

Dai et al. also found that FSP1 suppresses ferroptotic cell death and decreases the CoQ_10_ concentration following treatment with erastin, sorafenib, and RSL3 [5]. However, exogenous CoQ_10_ failed to reverse the ferroptotic cell death in FSP1 silencing cells. Strikingly, knockdown of FSP1 suppressed RSL3-induced charged multivesicular body protein 5 (CHMP5) and CHMP6 expression at the plasma membrane, and overexpression of CHMP5 rescued RSL3-, erastin-, and sorafenib-, induced cell death in both wild-type and FSP1 silencing cells. CHMP5 and CHMP6 are important subunits of the ESCRT-III-dependent membrane repair machinery, which is associated with ferroptosis resistance through membrane budding and fission. These results indicate that ESCRT-III-dependent membrane repair is another mechanism underlying FSP1-mediated ferroptosis resistance.

Recently, Mishima et al. identified three types of vitamin K (phylloquinone, menaquinone-4 (MK4), and menadione) that can protect cells from GPX4 deletion-induced ferroptosis, in a screen of naturally available vitamin compounds [1]. All three types of vitamin K rescued ferroptosis induced by ferroptosis inducers and glutamate-induced neuronal ferroptosis, but failed to block other types of cell death, including apoptosis, necroptosis, and proptosis. Mishima et al. further demonstrated that MK4 could prevent ferroptosis in vivo using two canonical mouse models. MK4 administration extended the survival time of *Alb*-creER^T2^; GPX4^fl/fl^ mice fed a vitamin E-deficient diet, and ameliorated liver injury in a mouse liver ischemia-reperfusion injury model. Subsequent research has demonstrated that vitamin K efficiently inhibits the propagation of lipid peroxides through a mechanism independent of its iron-chelating effect.

Vitamin K is a redox-active naphthoquinone that shares similar structural properties with ubiquinone, prompting Mishima et al. to evaluate whether FSP1 could catalyze the reduction of vitamin K. When recombinant human FSP1, NADH, and any of the three vitamin K were incubated in vitro, NADH was consumed and vitamin-hydroquinone was generated. These results demonstrated that FSP1 functions as a vitamin K reductase, producing its corresponding hydroquinone (VK-H_2_) to inhibit lipid peroxidation at the cost of NAD(P)H. As is the case for ubiquinone and ubiquinol, a-tocopherol could also be reproduced from its a-tocopheroxyl radical by VK-H_2_. N-myristylation of FSP1 seems to be important for its anti-ferroptotic role, because overexpression of wild-type, but not the myristylation-deficient FSP1 could rescue the anti-ferroptotic function of vitamin K in FSP1 knockout cells. These results were consistent with previous findings and highlighted the significance of N-myristylation of FSP1, which could target to the plasma membrane, induce NADH-dependent reduction of CoQ, and finally suppresses lipid peroxidation, the hallmark of ferroptosis. Protein N-myristoylation generally occurs co-translationally following cleavage of the initiator methionine by methionine aminopeptidase at the ribosome, or post-translationally following proteolytic cleavage of an N-terminal glycine residue by a protease. The specific molecular mechanisms of N-myristoylation of FSP1 requires further investigation. In addition, N-myristoyltransferase (NMT) is necessary for N-myristoylation. Strikingly, there is no expression of NMT in prokaryotes, an isozyme of NMT in lower eukaryotes, and two isozymes of NMT (NMT1 and NMT2) in most mammals. Ferroptosis is well-known as an evolutionarily conserved cell death in a variety of species, from prokaryotes to plants to mammals. Therefore, NMT may play a potential evolutionary role in ferroptosis resistance by mediating the N-myristoylation of FSP1.

An effective FSP1 inhibitor (iFSP1) has been identified through a counter-screen of FSP1-overexpressing cells in a wild-type or GPX4 knockout setting, based on the strong protective effect of FSP1 on GPX4 knockout cells [4]. Interestingly, FSP1-mediated enzymatic activity could be abrogated by iFSP1, but was resistant to warfarin, suggesting that FSP1 is the vitamin K reductase responsible for the warfarin-resistant alternative vitamin K reduction pathway in the canonical GGCX-VKOR-mediated cycle [1]. Consistently, MK4-treated FSP1^−/−^ mice converted MK4 to MK4 epoxide at a much lower rate and exhibited an extremely longer prothrombin time than FSP1^+/−^ mice when given high doses of warfarin, indicating antidotal FSP1 together with high-dose vitamin K averts warfarin poisoning. These findings suggest that FSP1 is a vitamin K reductase that maintains a warfarin-resistant non-canonical vitamin K cycle and inhibits ferroptosis by producing VK-H_2_ via NADH consumption to prevent lipid peroxidation. Notably, FSP1 activity varies across the human population. The effectiveness of warfarin was dampened in people with high FSP1 activity, while more vitamin K was required to protect against warfarin poisoning in people with low FSP1 activity. Besides, iFSP1 treatment have been proved to robustly sensitize cancer cells to ferroptosis [4]. The growth of ferroptosis-resistant H460 lung cancer cells can only be reduced by double knockout of GPX4 and FSP1, but not by GPX4 single knockout in a preclinical tumor xenograft mouse model [3]. Therefore, targeting FSP1 is a promising therapeutic strategy in these clinical backgrounds.

## Conclusion

**Fig. 1:**
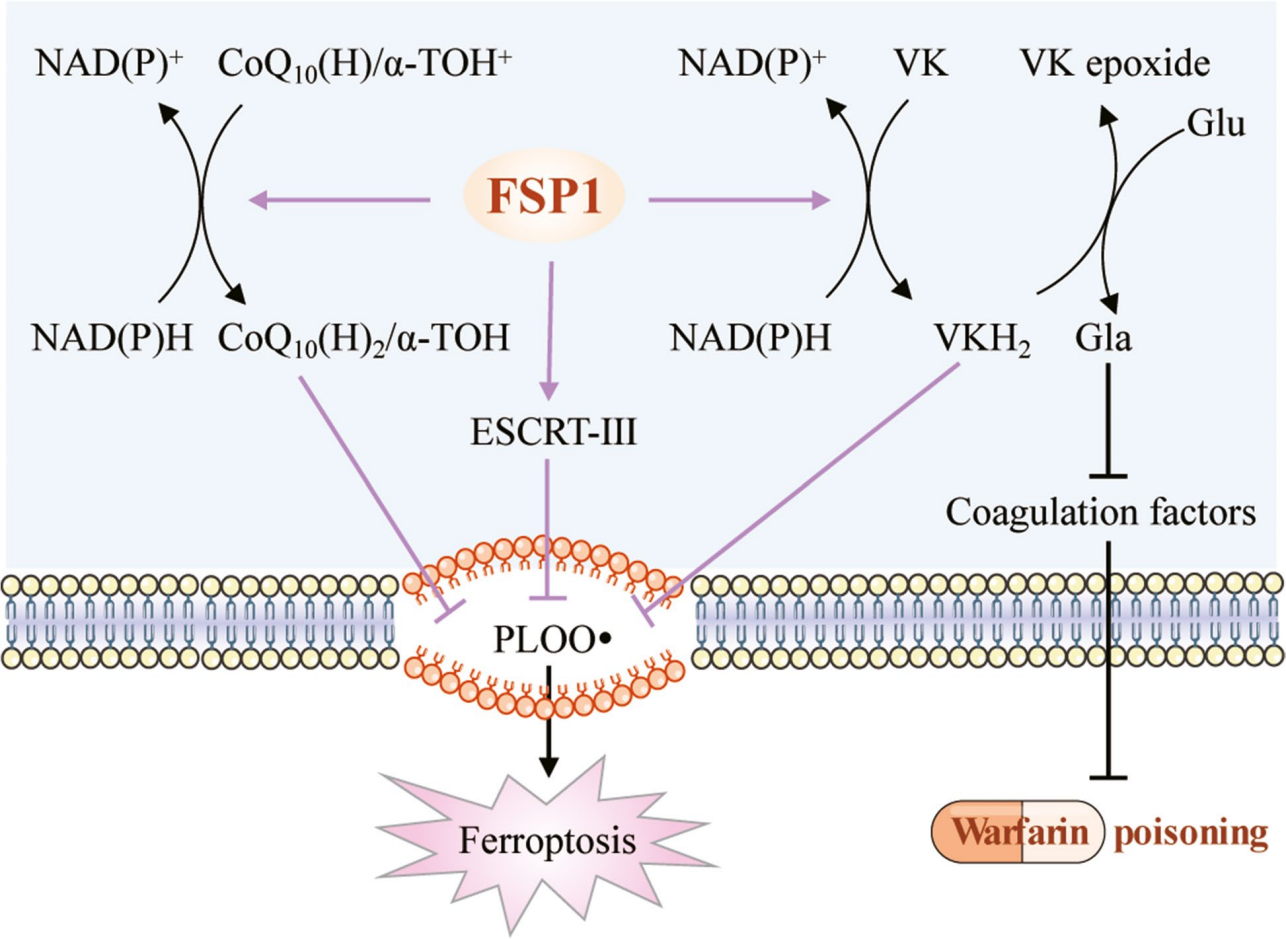
Suppression of ferroptosis by FSP1. Three different mechanisms of FSP1-mediated ferroptosis resistance have been uncovered: (1) the FSP1-CoQ_10_-NAD(P)H pathway, (2) the FSP1- ESCRT-III-dependent membrane repair pathway, and (3) the FSP1-VK-NAD(P)H pathway. Peroxyl radicals (PLOO•) initiates lipid radical-mediated autoxidation of lipid bilayers and finally induces ferroptosis

Since FSP1 was identified as an important ferroptosis suppressor protein, three different mechanisms of FSP1-mediated ferroptosis resistance have been uncovered: (1) the FSP1-CoQ_10_-NAD(P)H pathway, (2) the FSP1- ESCRT-III-dependent membrane repair pathway, and (3) the FSP1-VK-NAD(P)H pathway (Fig. 1). Given the critical role of FSP1 in ferroptosis resistance, it would be fascinating to determine its molecular structure and regulatory mechanism, and design FSP1 activators and inhibitors for clinical use.

## Data Availability

The datasets generated during and/or analysed during the current study are available from the corresponding author on reasonable request.
